# Abdominal Pain Associated With Celiac Artery Compression and Superior Mesenteric Artery (SMA) Syndrome: A Case Report

**DOI:** 10.7759/cureus.70520

**Published:** 2024-09-30

**Authors:** Aayush Lakkanna, Dilip Reddy, Basil Babu, Naveen Pentakota, Satish Subbiah Nagaraj

**Affiliations:** 1 General Surgery, Postgraduate Institute of Medical Education and Research, Chandigarh, IND

**Keywords:** celiac artery compression syndrome, dunbar syndrome, median arcuate ligament syndrome, postprandial abdominal discomfort, sma syndrome, superior mesenteric artery syndrome

## Abstract

Celiac artery compression syndrome (CACS) is an elusive cause of postprandial abdominal pain, commonly mistaken for conditions such as gall bladder disease. A 36-year-old male presented with postprandial abdominal pain. Imaging studies revealed compression of the celiac artery by the median arcuate ligament and an incidentally detected narrow aortomesenteric angle, indicating superior mesenteric artery (SMA) syndrome. The patient underwent laparoscopic release of the median arcuate ligament followed by celiac artery stenting to alleviate symptoms associated with CACS. This case highlights the coexistence of CACS and SMA syndrome in a single patient. In this case, the treatment was focused on addressing the symptoms of CACS, as the SMA syndrome did not significantly impact the patient's well-being. Thus, attention should be given to vascular phenomena when evaluating abdominal pain.

## Introduction

Celiac artery compression syndrome (CACS), also known as median arcuate ligament syndrome (MALS), is a condition characterized by the extrinsic compression of the celiac artery by the median arcuate ligament, a fibrous band of the diaphragm. This compression leads to postprandial epigastric pain, nausea, vomiting, and weight loss. The median arcuate ligament typically inserts at the level of the 12th thoracic or first lumbar vertebra. CACS can occur due to an abnormally high origin of the celiac artery or an abnormally low insertion of the diaphragm, often influenced by congenital factors. Patients with CACS often present with a history of postprandial abdominal discomfort, anorexia, and diarrhea, and they frequently have a history of ineffective treatment with proton pump inhibitors. Physical examination may reveal mild epigastric tenderness and an abdominal bruit [1].

Superior mesenteric artery (SMA) syndrome is a less common cause of vomiting and weight loss, resulting from the compression of the third part of the duodenum by the SMA, and is commonly seen in young adults. This compression is usually diagnosed via a contrast-enhanced CT (CECT) of the abdomen. The root causes of this condition are thought to include psychological factors, eating-related behaviors, and rapid weight loss [2,3]. This report presents the case of a 36-year-old male who was found to have both of these syndromes on cross-sectional imaging.

## Case presentation

A 36-year-old male presented to the emergency department (ED) with a one-and-a-half-year history of postprandial abdominal pain that was not relieved by proton pump inhibitors but was alleviated by analgesics. He had earlier visited multiple hospitals for the same complaints and was managed conservatively.

**Figure 1:**
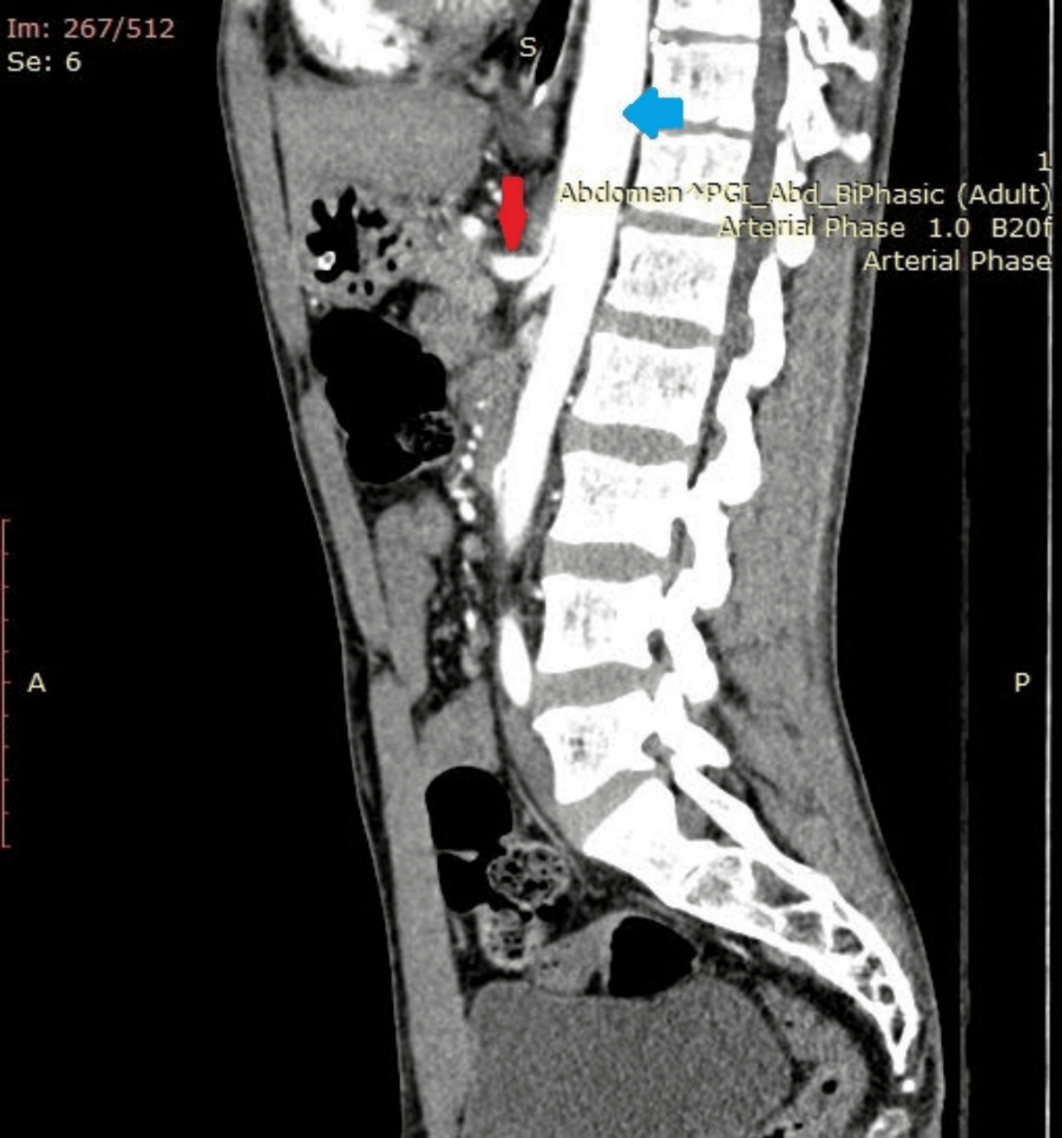
Preoperative contrast-enhanced CT scan of the abdomen (sagittal view) showing compressed celiac artery with post-stenotic dilation. Red arrow: celiac artery; Blue arrow: aorta; Yellow arrow: origin of superior mesenteric artery

**Figure 2:**
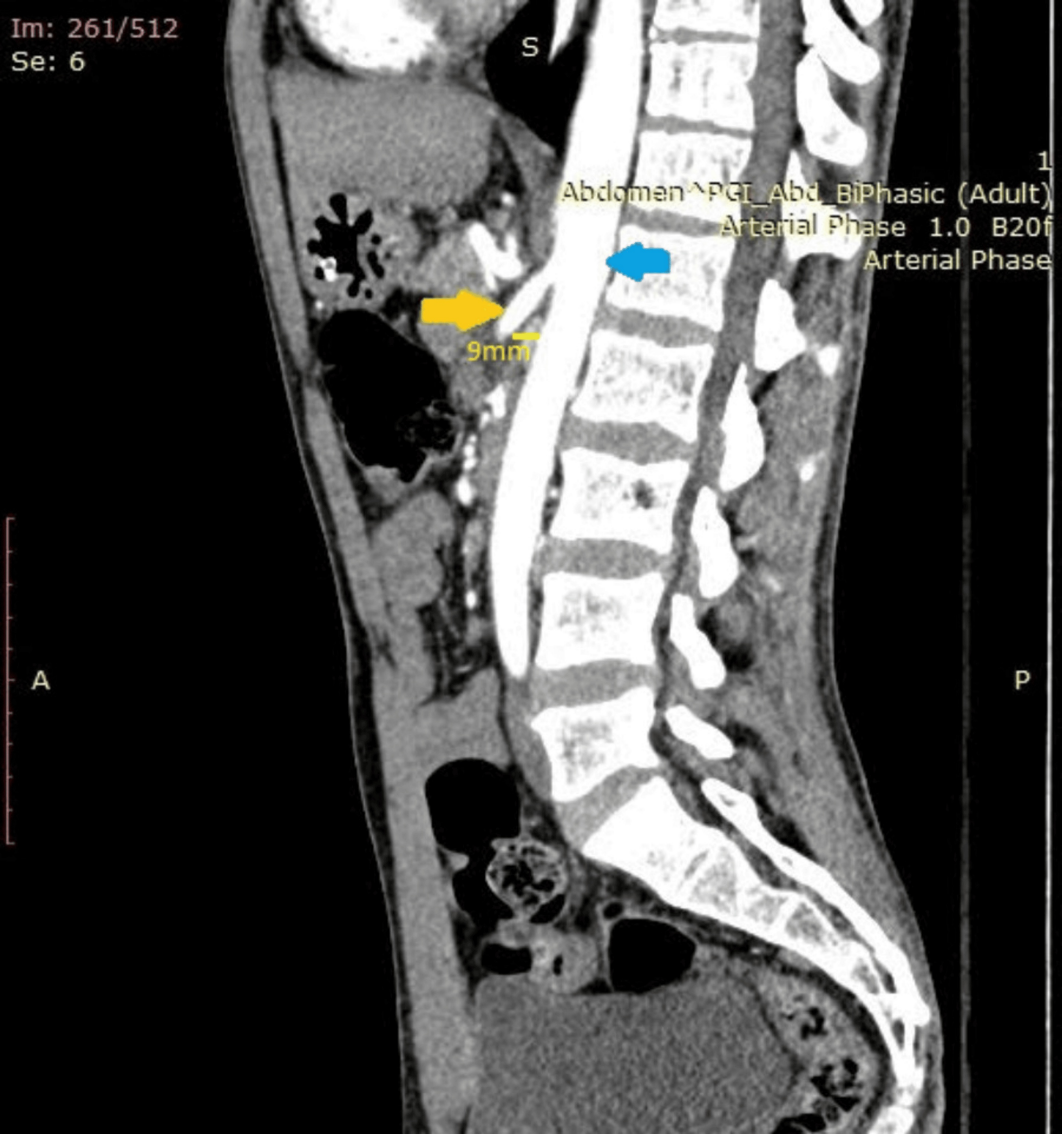
Preoperative contrast-enhanced CT scan of the abdomen (sagittal view) showing SMA syndrome, with reduced aortomesenteric distance (9 mm, marked in bright yellow) and aortomesenteric angle. Blue arrow: aorta; Yellow arrow: origin of SMA SMA: superior mesenteric artery

Abdominal examination was non-contributory and per rectal examination was normal. The urogenital examination was normal. Contrast-enhanced CT (CECT) of the abdomen, done in the ED, revealed focal kinking in the ostioproximal segment of the celiac artery, giving a J-shaped configuration, suggestive of MALS (Figure 1). The aortomesenteric angle was 19.7 degrees, and the aortomesenteric distance was 9 mm (Figure 2). A Doppler ultrasound revealed a celiac artery peak systolic velocity (PSV) of 80 cm/second. In the ED, he was evaluated and managed conservatively. The patient was discharged from the ED after his symptoms were relieved. His abdominal pain was attributed to MALS.

**Figure 3:**
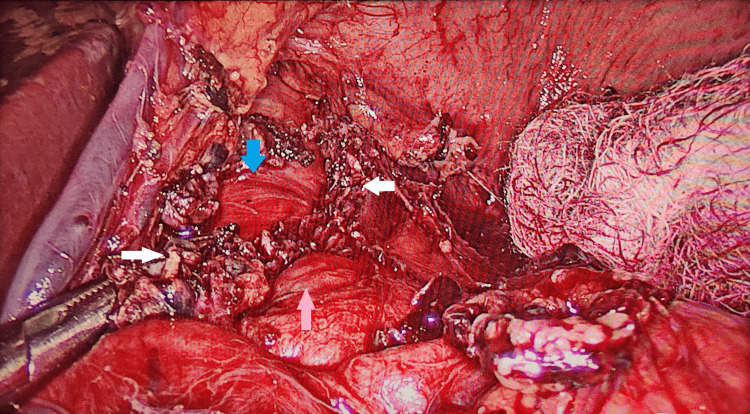
Intraoperative picture of laparoscopic median arcuate ligament release. Blue arrow: aorta; White arrows: fibers of median arcuate ligament; Pink arrow: celiac artery

Two months later, he was readmitted for definitive management. He underwent laparoscopic surgery, during which several fibers of the median arcuate ligament were found compressing the origin of the celiac trunk from the aorta (Figure 3). These fibers were released. Postoperatively, his abdominal pain did not resolve. A Doppler ultrasound of the celiac axis was performed, showing a celiac artery PSV of 74 cm/second, with no significant change during inspiration or expiration. Consequently, he underwent celiac artery stenting on postoperative day 10. Following the procedure, he was pain-free and discharged in a stable condition.

Follow-up and outcomes

**Figure 4:**
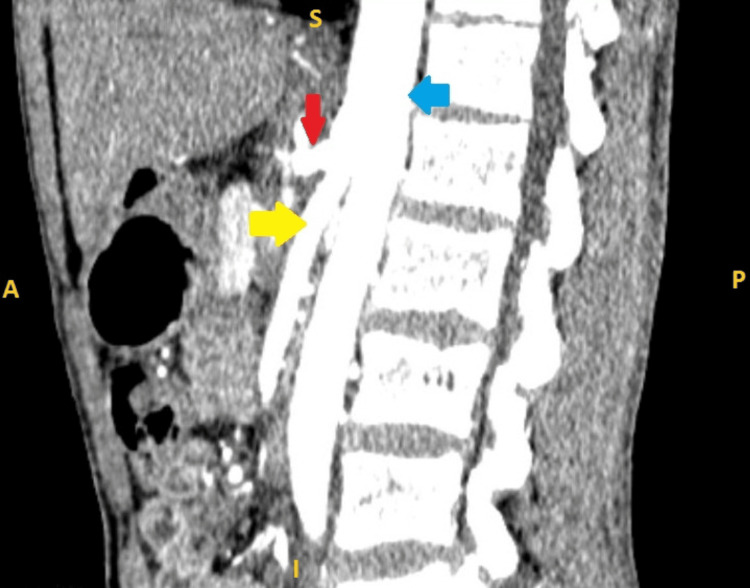
Postoperative contrast-enhanced CT scan of the abdomen (sagittal view) following celiac artery stenting. Red arrow: celiac artery; Yellow arrow: superior mesenteric artery; Blue arrow: aorta

**Figure 5:**
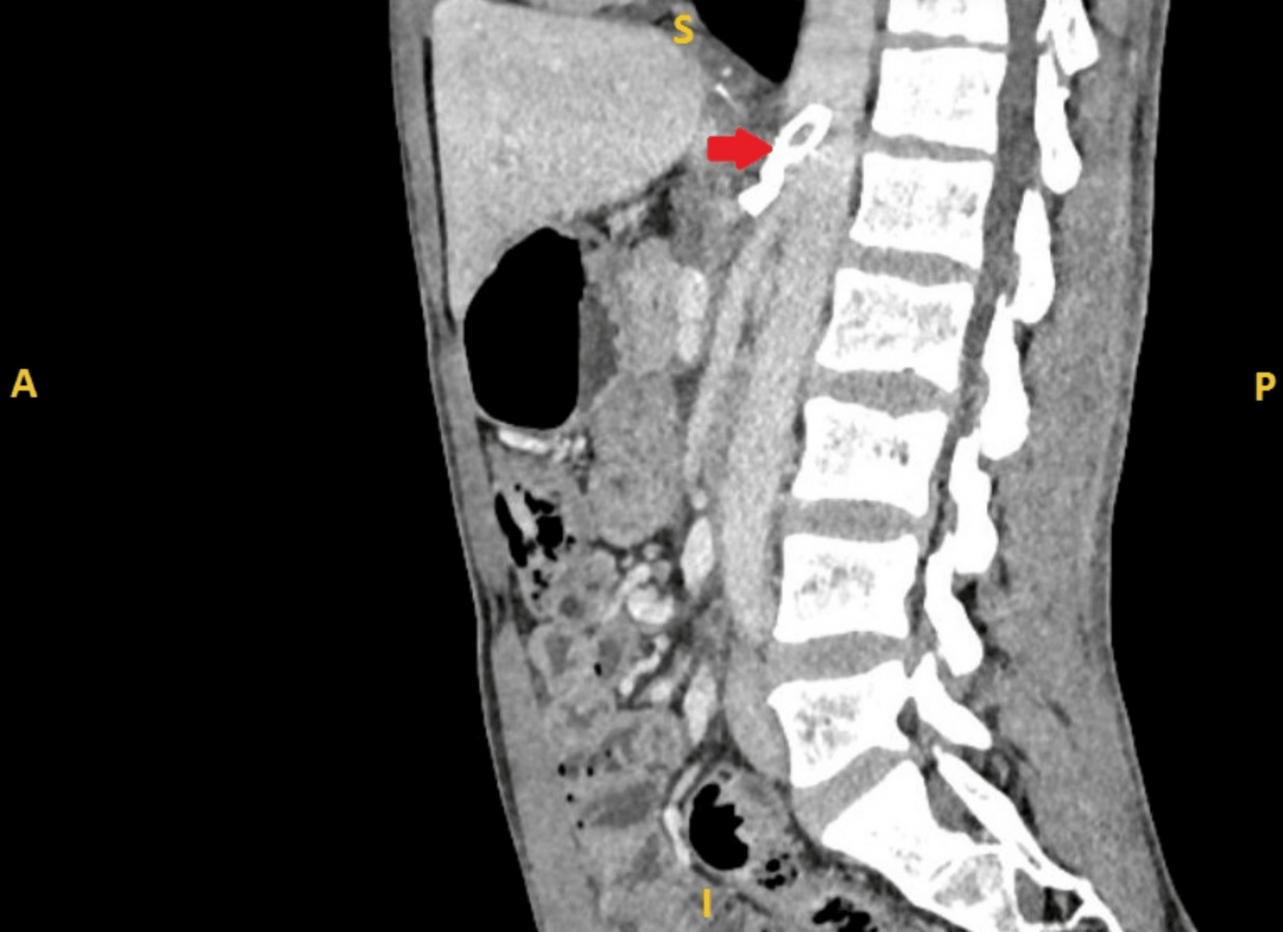
Postoperative contrast-enhanced CT scan of the abdomen (sagittal view) following celiac artery stenting showing stent in situ. Red arrow: celiac artery with stent in situ

The post-celiac artery stenting CECT abdomen scan (Figures 4-5) showed a patent celiac artery with good opacification. However, seven days after discharge, the patient experienced a recurrence of abdominal pain. He was initially managed with oral analgesics, and two months later, a CT-guided celiac ganglion block was performed. While this provided some relief, the pain recurred within a month. He was subsequently managed with oral analgesics. The patient has been on regular follow-up for the past few years, experiencing recurring episodes of pain, which have been managed with oral and intravenous (IV) analgesics.

## Discussion

The median arcuate ligament is a fibrous band that connects the two medial borders of the diaphragmatic crura. Compression of the celiac artery by the median arcuate ligament is believed to cause intermittent mesenteric ischemia. However, this may not fully explain the condition, as there is typically a rich collateral network of mesenteric vessels between the celiac artery and the SMA. Therefore, underlying celiac nerve plexus dysfunction may also be a contributory factor. Nerve dysfunction could lead to abnormal splanchnic vasoconstriction, resulting in ischemia [1].

The following criteria support the diagnosis of CACS on ultrasound: an expiratory peak velocity greater than 200 cm/second and a deflection angle greater than 50 degrees. Elevated celiac artery PSVs with deep expiration are also noted on ultrasonography. CT angiography (CTA) of the abdomen typically reveals compression of the celiac axis with focal stenosis and post-stenotic dilation in MALS [1].

In a study of postoperative patients with symptomatic celiac artery compression by Reilly et al., eight of 15 patients (53%) treated with celiac decompression alone remained asymptomatic at late follow-up, compared to 22 of 29 patients (76%) treated with celiac decompression plus some form of celiac revascularization [3]. Preoperative ganglion block and exercise gastric tonometry are useful diagnostic tools for predicting better outcomes after treatment [4].

Permanent celiac ganglion block, performed with neurolytic agents (alcohol or phenol), is used to relieve intractable pain in patients with malignant abdominal diseases (e.g., pancreatic, gastric, biliary, and metastatic cancers) or for chronic, severe visceral abdominal pain in patients with benign abdominal conditions [5].

Bech et al. described a case involving 27-year-old twin women with symptomatic mesenteric ischemia caused by median arcuate ligament compression [6]. Arteriography revealed severe celiac artery stenosis in one twin, celiac artery occlusion in the other, and proximal SMA narrowing with retrograde filling from a meandering mesenteric artery in both. Division of the ligament and direct celiac artery revascularization completely relieved symptoms in both patients.

Gander et al. reported a case of a 17-year-old male with recurrent postprandial abdominal pain [7]. CTA demonstrated focal stenosis at the origin of the celiac artery with post-stenotic dilation. Selective catheter angiography revealed focal stenosis of the celiac artery, accentuated by expiration and improved on inspiration. Doppler ultrasound also showed celiac artery stenosis with post-stenotic dilation and increased blood flow on expiration with improvement on inspiration. The patient was treated with laparoscopic surgical division of the median arcuate ligament [7].

SMA syndrome occurs due to duodenal compression by the SMA over the aorta. In a case series of SMA syndrome patients by Merrett et al., the primary anatomical feature observed was the narrowing of the angle between the SMA and the aorta, which is normally between 38° and 65° [2]. In their series, the angles ranged from 9° to 22°. This narrowing resulted in the compression of the third part of the duodenum as it crossed between the aorta and the SMA, and may have even compressed the left renal vein. The aortomesenteric distance was reduced from the normal 10-28 mm to 2-8 mm.

The patient discussed in the current report was treated for celiac artery compression only, as the incidentally discovered SMA syndrome features (aortomesenteric angle of 19.7° and aortomesenteric distance of 9 mm) did not impact his clinical well-being. He underwent minimally invasive surgery, followed by vascular stenting to prevent further episodes of abdominal angina. This report highlights the occurrence of multiple vascular anatomical abnormalities in a single patient, one of which had no demonstrable clinical effect.

## Conclusions

This case underscores the coexistence of two distinct vascular compression syndromes: CACS and incidentally detected SMA syndrome, in a single patient. The patient was primarily treated for celiac artery compression due to CACS, which was managed effectively with minimally invasive surgery followed by vascular stenting to prevent recurrent abdominal angina. Despite the radiologic evidence of SMA syndrome, characterized by a reduced aortomesenteric angle and distance, the absence of related clinical symptoms made further intervention unnecessary.

This report highlights the importance of thorough diagnostic evaluation when multiple vascular anomalies are present. The management approach should be guided by clinical manifestations rather than radiologic findings alone, as asymptomatic anomalies may not require intervention. The coexistence of CACS and radiologically detected SMA syndrome in this case emphasizes the complexity of vascular compression syndromes and the necessity for individualized treatment strategies.
